# Characteristic of 523 COVID-19 in Henan Province and a Death Prediction Model

**DOI:** 10.3389/fpubh.2020.00475

**Published:** 2020-09-08

**Authors:** Xiaoxu Ma, Ang Li, Mengfan Jiao, Qingmiao Shi, Xiaocai An, Yonghai Feng, Lihua Xing, Hongxia Liang, Jiajun Chen, Huiling Li, Juan Li, Zhigang Ren, Ranran Sun, Guangying Cui, Yongjian Zhou, Ming Cheng, Pengfei Jiao, Yu Wang, Jiyuan Xing, Shen Shen, Qingxian Zhang, Aiguo Xu, Zujiang Yu

**Affiliations:** ^1^Department of Respiration, The First Affiliated Hospital of Zhengzhou University, Zhengzhou, China; ^2^Department of Henan Gene Hospital, The First Affiliated Hospital of Zhengzhou University, Zhengzhou, China; ^3^Department of Infectious Diseases, The First Affiliated Hospital of Zhengzhou University, Zhengzhou, China; ^4^Department of Respiration, The Fifth Affiliated Hospital of Zhengzhou University, Zhengzhou, China; ^5^Department of Medical Services, The First Affiliated Hospital of Zhengzhou University, Zhengzhou, China; ^6^Department of Medical Information, The First Affiliated Hospital of Zhengzhou University, Zhengzhou, China

**Keywords:** novel coronavirus pneumonia, risk factors, death prediction model, random forest, epidemiology investigation

## Abstract

Certain high-risk factors related to the death of COVID-19 have been reported, however, there were few studies on a death prediction model. This study was conducted to delineate the clinical characteristics of patients with coronavirus disease 2019 (covid-19) of different degree and establish a death prediction model. In this multi-centered, retrospective, observational study, we enrolled 523 COVID-19 cases discharged before February 20, 2020 in Henan Province, China, compared clinical data, screened for high-risk fatal factors, built a death prediction model and validated the model in 429 mild cases, six fatal cases discharged after February 16, 2020 from Henan and 14 cases from Wuhan. Out of the 523 cases, 429 were mild, 78 severe survivors, 16 non-survivors. The non-survivors with median age 71 were older and had more comorbidities than the mild and severe survivors. Non-survivors had a relatively delay in hospitalization, with higher white blood cell count, neutrophil percentage, D-dimer, LDH, BNP, and PCT levels and lower proportion of eosinophils, lymphocytes and albumin. Discriminative models were constructed by using random forest with 16 non-survivors and 78 severe survivors. Age was the leading risk factors for poor prognosis, with AUC of 0.907 (95% CI 0.831–0.983). Mixed model constructed with combination of age, demographics, symptoms, and laboratory findings at admission had better performance (*p* = 0.021) with a generalized AUC of 0.9852 (95% CI 0.961–1). We chose 0.441 as death prediction threshold (with 0.85 sensitivity and 0.987 specificity) and validated the model in 429 mild cases, six fatal cases discharged after February 16, 2020 from Henan and 14 cases from Wuhan successfully. Mixed model can accurately predict clinical outcomes of COVID-19 patients.

## Introduction

In late December 2019, Wuhan City, Hubei Province, China found several cases of unexplained pneumonia. On January 7, 2020, a new coronavirus was detected in the laboratory and the whole genome sequence of the virus was obtained. On January 12, 2020, the World Health Organization temporarily named this new virus 2019 novel coronavirus (2019-nCoV). On February 11, 2020, the World Health Organization announced that the same time the International Virus Classification Committee named the new coronavirus “SARS-CoV-2.” Although the lethal rate of SARS-CoV-2 is not as high as SARS and MERS, it is more infectious than other viruses including influenza virus (1–3). The range of basic regeneration number (Ro) is estimated to be 2–5 (4, 5). China has effectively controlled the epidemic by adopting strict prevention and control measures, but in areas outside China, the epidemic of novel coronavirus is still spreading. The number of infections caused by SARS-CoV-2 is large and no specific therapeutic is available yet, which is the main cause of so many deaths. SARS-CoV-2 can cause pneumonia and systemic inflammation, leading to multiple organ failure in high-risk patients. More and more studies have focused on the high-risk factors of death. Demographic factors, advanced age, combined underlying diseases, and D-dimer exceeding 1 μg/L have been confirmed as risk factors for death in adult patients (6). In the absence of vaccines and specific antiviral drugs, targeted application of supportive therapy may be beneficial to relieve symptoms and protect organ functions (7). How to quickly identify high-risk patients in the early stage of the disease and actively adopt supportive treatment to reduce mortality is an urgent problem to be solved in the clinic. Cao Bin (6) and others reported some characteristics and clinical progress of the early stage of severe and dead patients, which improved our further understanding of the characteristics of dead patients. However, there are no relevant studies on the application of models to predict COVID-19 death. Using admission characteristics and laboratory test results to establish a predictive model can calculate the probability of over-all mortality due to SARS-CoV-2, identify high-risk patients as early as possible and give support to reduce mortality as soon as possible.

In this study, we collected data of 523 discharged cases of novel coronavirus infection in Henan Province, China and compared the demographics, clinical characteristics, laboratory test, imaging between the mild, severe survivors and non-survivors. We established a death prediction model using the data upon admission of the severe survivors and non-survivors.

## Methods

### Study Design and Participants

**Figure 1:**
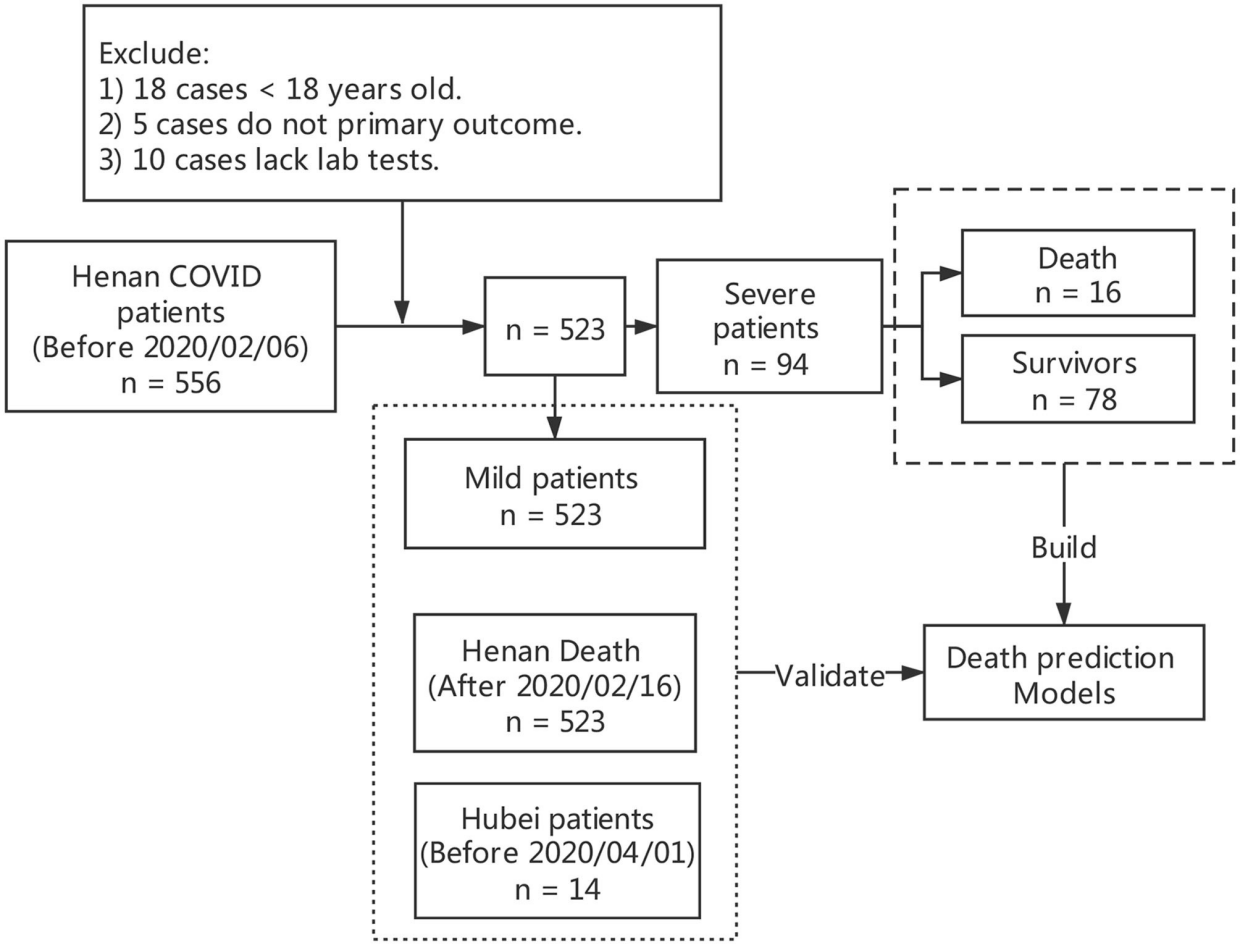
Flow diagram of included patients.

From January 22, 2020 to February 20, 2020, a total of 717 patients confirmed COVID-19 were discharged in 18 cities of Henan Province, China, of which 19 died. We designed a data collection table, including age, gender, epidemiological history, past history, clinical symptoms, laboratory examination, chest CT and recorded the treatment process and clinical outcome, and data of 556 patients with novel coronavirus pneumonia discharged before February 20, 2020 was collected. All data were checked by two physicians (AL and XM) and a third researcher (QZ) adjudicated any difference in interpretation between the two primary reviewers. For different interpretations and missing data, we contacted the doctor who filled out the form and the patient or their family members to review and supplement. Excluding 18 cases under the age of 18, 10 cases missing key information and five cases transferred to other hospitals with no end point, 523 cases were included for statistical analysis, of which 19 cases died including three fatal cases with data missing. According to the Guidance for Corona Virus Disease 2019 (6th edition) released by the National Health Commission of China, the enrolled cases were categorized as mild or severe (8). There were no deaths in the mild. According to the clinical outcome, we divided the severe into severe survivors and non-survivors. Up to April 1, there were 22 cases died of COVID-19 in Henan Province. We have managed to collect data of another six fatal cases of Henan Province and 14 cases from the Fourth People's Hospital of Wuhan to validate the predictive power of the model. The flow diagram of included patients is shown in Figure 1.

### Definition

The incubation period was defined as the interval between the potential earliest date of contact of the transmission source (wildlife or person of suspected or confirmed case) and the potential earliest date of symptom onset (i.e., cough, fever, fatigue, or myalgia). We excluded cases with an incubation period of <1 day or cases of continuous exposure, because those patients continued to be infected. Fever was defined as an axillary temperature of 37.3°C or higher. Lymphopenia was defined as a lymphocyte count of <1,200 per cubic millimeter. Thrombocytopenia was defined as a platelet count of <100,000 per cubic millimeter. Chest CT was divided into normal, mild, moderate and severe infections according to the range of lesions. The range of lesions <15% was mild; the range of lesions 15–40% was moderate; the range of lesions > 40% was severe.

### Statistical Analysis

Statistical analyses on cohort characteristics were performed on R version 3.6.1. Participants' demographic, laboratory findings and questionnaire were summarized with a standardized statistical significance test method, categorical variables were shown as counts and percentages [*n* (%)], and associations were tested using a fisher' exact test. Continuous variables were shown as median (interquartile range, IQR), and differences between groups were analyzed with non-parametric test (Wilcoxon's rank-sum test). A single-sided *p* < 0.05 was considered statistically significant. Discriminative models were constructed by using random forest with leave-one-out cross validation, features were selected by using embedded backward selection. Missing data were filled by chose median value in relative cohort (Severe death, severe survival, and mild) for model construction and validation. Receiver operating characteristic (ROC) curve and Precision-Recall curve were visualized by using R program package “pROC” and “precrec,” respectively.

## Results

### Clinical Characteristics of the Study Patients According to Disease Severity and Clinical Outcome in Severe

Table 1 shows that among the 523 patients 429/523 (82.03%) were mild, 94/523 (17.97%) severe, and 16/94 (17.02%) in severe cases died of COVID-19. The median age of the 523 patients was 44.0 years (IQR 32–54), with male patients (55.26%) accounting for the majority. Severe patients were older than mild patients (50.00 [IQR 38.25–61.5] vs. 42 [IQR 31–54]), and non-survivors were older than 65 years with a median age of 71 years (IQR 67.75–80). Age difference between the cases was statistically significant.

**Table 1:** Clinical characteristics of the study patients according to disease severity and clinical outcome in severe.

**Characteristics**	**All patients (*N* = 523)**	**Disease severity**			**Clinical outcome in severe**		
		**Mild (*N* = 429)**	**Severe (*N* = 94)**	***p*-value**	**Non-survivors (*N* = 16)**	**Survivors (*N* = 78)**	***p*-value**
Age, years	44 (32–54)	42 (31–54)	50 (38.25–61.5)	0.00004	71 (67.75–80)	47 (34–54.75)	0
18–49	314/523 (60.04)	270/429 (62.94)	44/94 (46.81)	0.00384	1/16 (6.25)	43/78 (55.13)	0.00036
50–64	146/523 (27.92)	121/429 (28.21)	25/94 (26.6)	0.75274	1/16 (6.25)	24/78 (30.77)	0.03505
≥65	46/523 (8.8)	23/429 (5.36)	23/94 (24.47)	0	14/16 (87.5)	9/78 (11.54)	0
Female sex	234/523 (44.74)	199/429 (46.39)	35/94 (37.23)	0.06599	3/16 (18.75)	32/78 (41.03)	0.07824
**Exposure to Source of Transmission Within Past 14 Days**							
Had the history of travel or residence in Wuhan and its surrounding areas, or other communities where the case of COVID-19 had been reported	324/523 (61.95)	272/429 (63.4)	52/94 (55.32)	0.08999	5/16 (31.25)	47/78 (60.26)	0.03216
Had contact with Wuhan residents	158/523 (30.21)	132/429 (30.77)	26/94 (27.66)	0.32199	6/16 (37.5)	20/78 (25.64)	0.24972
Cluster	76/523 (14.53)	66/429 (15.38)	10/94 (10.64)	0.15317	2/16 (12.5)	8/78 (10.26)	0.5381
Had contact with patients confirmed0020COVID-19	136/523 (26)	115/429 (26.81)	21/94 (22.34)	0.22412	5/16 (31.25)	16/78 (20.51)	0.263
Not clear	67/523 (12.81)	48/429 (11.19)	19/94 (20.21)	0.01702	3/16 (18.75)	16/78 (20.51)	0.58924
**Comorbidity**							
COPD	13/463 (2.81)	6/373 (1.61)	7/90 (7.78)	0.00526	5/15 (33.33)	2/75 (2.67)	0.00117
Asthma	3/459 (0.65)	2/369 (0.54)	1/90 (1.11)	0.48126	0/15 (0)	1/75 (1.33)	0.83333
Interstitial pneumonia	8/462 (1.73)	7/372 (1.88)	1/90 (1.11)	0.51749	1/15 (6.67)	0/75 (0)	0.16667
Diabetes	42/462 (9.09)	29/371 (7.82)	13/91 (14.29)	0.0475	5/15 (33.33)	8/76 (10.53)	0.03604
Coronary heart disease	24/461 (5.21)	13/371 (3.5)	11/90 (12.22)	0.00237	5/15 (33.33)	6/75 (8)	0.01681
Hypertension	74/465 (15.91)	48/375 (12.8)	26/90 (28.89)	0.00034	7/15 (46.67)	19/75 (25.33)	0.09088
Cerebral infarction	11/461 (2.39)	9/371 (2.43)	2/90 (2.22)	0.63343	1/15 (6.67)	1/75 (1.33)	0.30712
Cerebral hemorrhage	6/459 (1.31)	4/369 (1.08)	2/90 (2.22)	0.33495	1/15 (6.67)	1/75 (1.33)	0.30712
Cancer	7/230 (3.04)	4/191 (2.09)	3/39 (7.69)	0.09676	2/10 (20)	1/29 (3.45)	0.15593
Pregnancy	2/469 (0.43)	2/382 (0.52)	0/87 (0)	0.66309	0/16 (0)	0/71 (0)	1
Digestive system disease	11/307 (3.58)	10/257 (3.89)	1/50 (2)	0.44053	0/10 (0)	1/40 (2.5)	0.8
Chronic kidney disease	5/307 (1.63)	3/256 (1.17)	2/51 (3.92)	0.19413	0/11 (0)	2/40 (5)	0.61176
**Symptoms**							
Fever	449/506 (88.74)	360/412 (87.38)	89/94 (94.68)	0.02648	15/16 (93.75)	74/78 (94.87)	0.6154
Fatigue	190/480 (39.58)	151/387 (39.02)	39/93 (41.94)	0.34364	10/16 (62.5)	29/77 (37.66)	0.06087
Cough	309/496 (62.3)	241/403 (59.8)	68/93 (73.12)	0.01064	11/16 (68.75)	57/77 (74.03)	0.43858
Dyspnea	41/471 (8.7)	18/378 (4.76)	23/93 (24.73)	0	7/16 (43.75)	16/77 (20.78)	0.05703
Gasp	44/468 (9.4)	22/375 (5.87)	22/93 (23.66)	0	4/16 (25)	18/77 (23.38)	0.55775
Chest tightness	92/471 (19.53)	60/378 (15.87)	32/93 (34.41)	0.0001	11/16 (68.75)	21/77 (27.27)	0.00236
Nasal congestion	32/467 (6.85)	20/374 (5.35)	12/93 (12.9)	0.01303	1/16 (6.25)	11/77 (14.29)	0.34485
Runny nose	31/467 (6.64)	21/374 (5.61)	10/93 (10.75)	0.06599	1/16 (6.25)	9/77 (11.69)	0.45525
Sore throat	65/470 (13.83)	51/378 (13.49)	14/92 (15.22)	0.38807	2/16 (12.5)	12/76 (15.79)	0.54396
Expectoration	138/480 (28.75)	107/387 (27.65)	31/93 (33.33)	0.16827	7/16 (43.75)	24/77 (31.17)	0.24499
Anorexia	61/473 (12.9)	44/380 (11.58)	17/93 (18.28)	0.06352	5/16 (31.25)	12/77 (15.58)	0.13271
Diarrhea	25/467 (5.35)	17/374 (4.55)	8/93 (8.6)	0.10097	2/16 (12.5)	6/77 (7.79)	0.41557
Headache	37/471 (7.86)	27/378 (7.14)	10/93 (10.75)	0.17119	0/16 (0)	10/77 (12.99)	0.13578
Dizziness	28/470 (5.96)	23/377 (6.1)	5/93 (5.38)	0.50991	0/16 (0)	5/77 (6.49)	0.38017
Muscle and joint pain	39/464 (8.41)	26/371 (7.01)	13/93 (13.98)	0.02981	1/16 (6.25)	12/77 (15.58)	0.29732
**The Basic Vital Signs on Admission**							
Respiratory rate >24 breaths per min	4/489 (0.82)	3/399 (0.75)	1/90 (1.11)	0.02936	1/13 (7.69)	0/77 (0)	0.00554
Pulse oxygen saturation <90%	8/165 (4.85)	0/122 (0)	8/43 (18.6)	0	5/11 (45.45)	3/32 (9.38)	0.00019
Fever on admission, °C	37.2 (36.7-37.9)	37.1 (36.7-37.9)	37.2 (36.8-38)	0.26312	36.7 (36.6-37.1)	37.3 (36.8-38)	0.00776
<37.5	293/496 (59.07)	237/404 (58.66)	56/92 (60.87)	0.68771	12/15 (80)	44/77 (57.14)	0.09703
37.5–38.0	90/496 (18.15)	78/404 (19.31)	12/92 (13.04)	0.15947	1/15 (6.67)	11/77 (14.29)	0.37752
38.1–39.0	97/496 (19.56)	77/404 (19.06)	20/92 (21.74)	0.55865	1/15 (6.67)	19/77 (24.68)	0.10886
>39.0	16/496 (3.23)	12/404 (2.97)	4/92 (4.35)	0.34306	1/15 (6.67)	3/77 (3.9)	0.51568
Time form illness onset to seeing a doctor, days	2 (0–5)	2 (0–5)	2 (1–5)	0.41842	4.5 (1.75–7)	2 (1–4)	0.04202
Time form illness onset to hospital admission, days	4 (2–7)	4 (2–7)	3 (2–7)	0.29878	8 (6–10)	3 (1–6)	0.00047
Incubation period, days	5 (1–9)	5 (1–9)	4 (1–9)	0.30537	5 (2–10)	4 (1–8)	0.10345

*Data are median (IQR)or n/N (%). P-values were calculated by Fisher's exact test or Mann-Whitney U-test. COPD, chronic obstructive pulmonary disease*.

Within 14 days before the onset, 324 (61.95%) had lived in or visited Wuhan; 158 (30.21%) had contact history with Wuhan returnees; 136 (26%) had confirmed contact history of COVID-19 cases; 76 (14.53%) occurred from familial clusters, and 67 (12.81%) had unknown contact history. Non-survivors were not much different from the mild or severe survivors in terms of epidemiological history.

Hypertension (74/465 [15.91%]), diabetes (42/462 [9.09%]), coronary heart disease (24/461 [5.21%]) were the most common comorbidities. The average number of comorbid diseases in the non-survivors was 1.94 which was significantly higher than that of the mild and severe survivors. The most common symptoms on admission were fever (449 [88.74%], cough (309 [62.3%] and fatigue (190 [39.58%]); the more common symptoms were expectoration (138 [28.75%]), chest tightness (92 [19.53%]), sore throat (65 [13.83%]), anorexia (61 [12.9%]), gasp (44 [9.4%]), and dyspnea (41 [8.7 %]). Muscle and joint pain, runny nose, diarrhea, dizziness, and headache were rare. The symptoms of fever, cough, dyspnea, gasp, chest tightness, nasal congestion, and muscle and joint pain had a higher incidence in severe cases, and the difference was significant; the incidence of chest tightness in non-survivors was higher than that in severe survivors. The patients in the non-survivors had more symptoms at the onset.

Four (0.82%) had a respiratory rate > 24 breaths/min, one of them died; 8 (4.85%) pulse oxygen saturation < 90%, all severe; median body temperature 37.2°C (IQR 36.7–37.9), 293 (59.07%) body temperature < 37.5°C, 16 (3.23%) body temperature > 39°C and 80% non-survivors body temperature < 37.5°C upon admission.

The median duration from onset of symptoms to first visit to doctor was 2 days (IQR 0–5), from onset of symptoms to first hospitalization 4 days (IQR 2–7) while 8 days (IQR 6–10) in non-survivors. The median incubation period was 5 days (IQR 1–9), with no significant difference between the cases.

### Radiographic and Laboratory Findings on Admission

Table 2 shows the imaging and laboratory examination results. Of all the cases, 419 patients had detailed chest CT data on initial admission, with 17 (4.06%) being normal; 224 (53.46%) chest CT lesions < 15%; 154 (36.75%) chest CT lesions between 15 and 40%; 24 (5.73%) chest CT lesions > 40%, of which 15 were severe. In the non-survivors, 100% of patients had a chest CT lesion area of more than 15% for the first time.

**Table 2:** Radiographic and laboratory findings on admission.

**Characteristics**	**All patients (*N* = 523)**	**Disease severity**			**Clinical outcome in severe**		
		**Mild (*N* = 429)**	**Severe (*N* = 94)**	***p*-value**	**Non-survivors (*N* = 16)**	**Survivors (*N* = 78)**	***p*-value**
**Radiographic Findings**							
**Chest CT**							
Normal	17/419 (4.06)	13/342 (3.8)	4/77 (5.19)	0.38245	0/10 (0)	4/67 (5.97)	0.56639
Mild	224/419 (53.46)	202/342 (59.06)	22/77 (28.57)	0	0/10 (0)	22/67 (32.84)	0.02666
Moderate	154/419 (36.75)	118/342 (34.5)	36/77 (46.75)	0.04398	6/10 (60)	30/67 (44.78)	0.2873
Severe	24/419 (5.73)	9/342 (2.63)	15/77 (19.48)	0	4/10 (40)	11/67 (16.42)	0.09699
**Laboratory Findings**							
**Pathogens identified**							
COVID-19 viral nucleic acid test positive on the first time	323/495 (65.25)	258/404 (63.86)	65/91 (71.43)	0.10514	16/16 (100)	49/75 (65.33)	0.00249
Influenza A virus Ag+	8/323 (2.48)	8/263 (3.04)	0/60 (0)	0.18936	0/7 (0)	0/53 (0)	1
Influenza B virus Ag+	13/324 (4.01)	10/264 (3.79)	3/60 (5)	0.44419	1/7 (14.29)	2/53 (3.77)	0.31543
Mycoplasma pneumonia IgM Ab+	28/319 (8.78)	22/262 (8.4)	6/57 (10.53)	0.3824	1/7 (14.29)	5/50 (10)	0.5621
HBsAg+	26/357 (7.28)	23/288 (7.99)	3/69 (4.35)	0.2214	0/11 (0)	3/58 (5.17)	0.58892
HCV-Ab+	7/353 (1.98)	7/285 (2.46)	0/68 (0)	0.22041	0/11 (0)	0/57 (0)	1
TP-Ab+	7/345 (2.03)	7/277 (2.53)	0/68 (0)	0.21186	0/11 (0)	0/57 (0)	1
**Blood routine**							
Leucocyte count, × 10^9^ /L	4.82 (3.585–6.225)	4.74 (3.5025–6.0475)	5.38 (4–7.05)	0.00281	8.66 (7–12.335)	5.12 (3.8675–5.9875)	0.00007
>10	24/487 (4.93)	14/398 (3.52)	10/89 (11.24)	0.00537	5/15 (33.33)	5/74 (6.76)	0.01073
4–10	310/487 (63.66)	252/398 (63.32)	58/89 (65.17)	0.74263	9/15 (60)	49/74 (66.22)	0.64496
<4	153/487 (31.42)	132/398 (33.17)	21/89 (23.6)	0.07869	1/15 (6.67)	20/74 (27.03)	0.07944
Platelet count, ×10^9^ /L	175.5 (143–210)	176 (143–209.5)	168 (145–216)	0.29403	153 (93.25–210.75)	171 (147–213.5)	0.08684
<100	26/452 (5.75)	19/375 (5.07)	7/77 (9.09)	0.13423	4/14 (28.57)	3/63 (4.76)	0.01824
Absolute lymphocyte count, ×10^9^ /L	1.1 (0.84–1.49)	1.12 (0.9–1.55)	0.92 (0.595–1.225)	0.00005	0.72 (0.44–0.89)	0.985 (0.6325–1.2475)	0.03876
<0.8	98/448 (21.88)	67/369 (18.16)	31/79 (39.24)	0.00004	7/13 (53.85)	24/66 (36.36)	0.23802
Lymphocyte percentage, %	24.5 (17.055–33.18)	24.8 (18.575–33.625)	19.75 (9.36–29.025)	0.00029	8.34 (5.3–13.405)	21.75 (11.225–29.775)	0.00526
<20	152/456 (33.33)	112/376 (29.79)	40/80 (50)	0.0005	11/14 (78.57)	29/66 (43.94)	0.01858
Absolute neutrophil count, ×10^9^ /L	3.09 (2.085–4.415)	3.04 (2.06–4.3)	3.73 (2.37–5.765)	0.01557	6.29 (4.03–11.09)	3.54 (2.25–4.62)	0.01067
Neutrophil percentage, %	65.5 (56.35–74.65)	65.4 (56.5–73.7)	68.6 (56.6–85.1)	0.00649	86.105 (80.1125–92.025)	65.95 (55.4–77.1775)	0.00172
Absolute eosinophil count, ×10^9^ /L	0.01 (0–0.03)	0.01 (0–0.03)	0.01 (0–0.03)	0.24402	0 (0–0.02)	0.01 (0–0.03)	0.15484
<0.2	375/388 (96.65)	314/324 (96.91)	61/64(95.31)	0.36519	10/11 (90.91)	51/53 (96.23)	0.43774
Eosinophil percentage, %	0.2 (0–0.7)	0.2 (0–0.78)	0.1 (0–0.4)	0.00506	0 (0–0.2)	0.1 (0–0.4)	0.0478
<0.1	143/405 (35.31)	109/330 (33.03)	34/75 (45.33)	0.04418	9/15 (60)	25/60 (41.67)	0.20205
<0.5	276/405 (68.15)	217/330 (65.76)	59/75 (78.67)	0.03031	13/15 (86.67)	46/60 (76.67)	0.32384
**Hemagglutination examination**							
Fibrinogen, g/L	3.48 (2.8–4.76)	3.43 (2.79–4.72)	3.607 (2.99–5.075)	0.18678	3.7055 (2.685–5.035)	3.607 (2.99–5.0825)	0.42719
>4	127/367 (34.6)	97/297 (32.66)	30/70 (42.86)	0.10666	5/14 (35.71)	25/56 (44.64)	0.54597
Prothrombin time, s	11.9 (10.7–12.9)	11.9 (10.77–12.9)	11.8 (10.6–13)	0.41355	12.5 (11.25–14.6)	11.7 (10.5–12.8)	0.06903
≥16	21/370 (5.68)	17/302 (5.63)	4/68 (5.88)	0.56081	1/11 (9.09)	3/57 (5.26)	0.51496
D-Dimer, mg/L	0.41 (0.18–1.77225)	0.39 (0.16–0.98)	0.8 (0.19–4.18)	0.04076	6.9 (1–32.47)	0.46 (0.1625–1.63225)	0.00199
0.5–1	57/304 (18.75)	47/241 (19.5)	10/63 (15.87)	0.54445	2/13 (15.38)	8/50 (16)	0.66288
≥1	87/304 (28.62)	60/241 (24.9)	27/63 (42.86)	0.00498	10/13 (76.92)	17/50 (34)	0.00534
**Liver function examination**							
Glutamic-pyruvic transaminase, U/L	22.45 (15–35)	22 (15–34)	26.5 (19.375–38.825)	0.00972	28 (21–41.4)	26 (19.3–38.1)	0.38532
>40	86/378 (22.75)	69/310 (22.26)	17/68 (25)	0.62525	3/11 (27.27)	14/57 (24.56)	0.55735
Glutamic-oxalacetic transaminase, U/L	25 (19–33)	24 (18.8275–31.875)	30 (20.75–38.075)	0.00755	37 (32–42.25)	27 (20–35.4)	0.01613
>40	55/374 (14.71)	40/306 (13.07)	15/68 (22.06)	0.09858	5/11 (45.45)	10/57 (17.54)	0.05567
Total bilirubin, μmol/L	10.25 (7.575–15.35)	10.2 (7.5–14.7)	11.18 (8.05–17)	0.09421	14.755 (8.975–17.505)	10.9 (8.05–16.2)	0.16849
>17.1	61/384 (15.89)	45/311 (14.47)	16/73 (21.92)	0.11717	4/12 (33.33)	12/61 (19.67)	0.24509
Lactate dehydrogenase, U/L	208 (170.69–255.445)	202.38 (166.9–235)	246.5 (186.75–387.795)	0	437 (306.315–715.5)	223 (177.4–343.74)	0.00182
≥250	102/383 (26.63)	64/307 (20.85)	38/76 (50)	0	9/11 (81.82)	29/65 (44.62)	0.02248
Creatine Kinase, U/L	69.66 (45–113)	69 (43–106.81)	72.5 (53.99–168.3125)	0.0169	172.875 (119.75–282.84)	67.195 (53–113.75)	0.01175
≥200	36/363 (9.92)	22/299 (7.36)	14/64 (21.88)	0.00042	4/10 (40)	10/54 (18.52)	0.13805
Albumin, g/L	40.4 (36.7–44)	40.7 (37.2–44.15)	39.9 (34–42.8)	0.00594	32.8 (29.75–39.45)	39.95 (34.975–43.175)	0.01982
<30	17/380 (4.47)	8/311 (2.57)	9/69 (13.04)	0.00094	3/11 (27.27)	6/58 (10.34)	0.14825
**Renal function examination**							
Creatinine, μmol/L	65.29 (54–78.35)	65.29 (54–78.1)	64.85 (54.775–79.55)	0.47011	83.5 (61.38–88)	63 (54.85–71)	0.0379
≥133	5/354 (1.41)	3/284 (1.06)	2/70 (2.86)	0.25749	2/11 (18.18)	0/59 (0)	0.02277
Blood urea nitrogen, mmol/L	3.94 (3.035–5.17)	3.905 (2.9925–5.03)	4.2 (3.2–6.9625)	0.08176	5.695 (4.2075–9.99)	3.815 (3.175–5.5725)	0.07124
>8	49/332 (14.76)	35/266 (13.16)	14/66 (21.21)	0.09869	4/10 (40)	10/56 (17.86)	0.12549
Glomerular filtration rate, ml/min/1.73 m^2^	106.39 (89.12–120.9885)	106.49 (88.239–120.9885)	105.0085 (89.6875–121.645)	0.48903	114 (106.59–122.84)	104.17 (88.48–118.3)	0.35179
>120	16/55 (29.09)	12/39 (30.77)	4/16 (25)	0.46779	1/3 (33.33)	3/13 (23.08)	0.60714
**Arterial blood gas analysis**							
PH value	7.435 (7.41–7.47)	7.43 (7.409–7.46)	7.45 (7.42625–7.48)	0.02645	7.4475 (7.425–7.4675)	7.455 (7.4285–7.48)	0.29188
>7.45	41/97 (42.27)	21/61 (34.43)	20/36 (55.56)	0.04183	5/10 (50)	15/26 (57.69)	0.48092
7.35–7.45	54/97 (55.67)	39/61 (63.93)	15/36 (41.67)	003294	4/10 (40)	11/26 (42.31)	0.60234
<7.35	2/97 (2.06)	1/61 (1.64)	1/36 (2.78)	0.60696	1/10 (10)	0/26 (0)	0.27778
PCO_2_, mmHg	35.7 (31.7–39)	36.3 (32–39.2)	35 (30.775–38.45)	0.0975	32.5 (26.4–35.55)	35.05 (31.85–38.55)	0.10487
<35	40/97 (41.24)	23/61 (37.7)	17/36 (47.22)	0.35762	7/10 (70)	10/26 (38.46)	0.09239
35–45	51/97 (52.58)	34/61 (55.74)	17/36 (47.22)	0.41712	2/10 (20)	15/26 (57.69)	0.04698
>45	6/97 (6.19)	4/61 (6.56)	2/36 (5.56)	0.60563	1/10 (10)	1/26 (3.85)	0.48413
PO_2_, mmHg	80.8 (67–96.6)	86.9 (78.8–103)	68.5 (53.8–83.65)	0.00008	62.6 (49.275–86.45)	68.5 (58.575–81.5)	0.2567
<60	17/97 (17.53)	5/61 (8.2)	12/36 (33.33)	0.00166	5/10 (50)	7/26 (26.92)	0.17793
**Other examination**							
Brain natriuretic peptide, pg/mL	117.8 (53.5625–604.9)	90.71 (41–109)	612.5 (256–1121)	0	455.95 (314.715–666.75)	736.4 (256–1166)	0.22236
>100	46/74 (62.16)	17/41 (41.46)	29/33 (87.88)	0.00004	8/8 (100)	21/25 (84)	0.30914
C-reactive protein, mg/L	11.425 (4.075–28)	10 (3.45–22.715)	30.86 (11–73.49)	0	68 (30.91–96)	21.105 (10.6175–50.175)	0.02518
>10	211/372 (56.72)	152/303 (50.17)	59/69 (85.51)	0	12/13 (92.31)	47/56 (83.93)	0.39436
Procalcitonin, ng/mL	0.07 (0.05–0.1525)	0.055 (0.05–0.11)	0.13 (0.05925–0.20475)	0.00012	0.1835 (0.15625–0.3225)	0.12 (0.05225–0.20475)	0.04056
<0.1	132/235 (56.17)	116/187 (62.03)	16/48 (33.33)	0.00035	0/8 (0)	16/40 (40)	0.02787
0.1–0.25	63/235 (26.81)	41/187 (21.93)	22/48 (45.83)	0.00085	6/8 (75)	16/40 (40)	0.07686
0.25–0.5	25/235 (10.64)	20/187 (10.7)	5/48 (10.42)	0.95548	0/8 (0)	5/40 (12.5)	0.38428
≥0.5	15/235 (6.38)	10/187 (5.35)	5/48 (10.42)	0.16861	2/8 (25)	3/40 (7.5)	0.18874
Cardiac troponin T, μg/L	0.01 (0.004–0.0895)	0.01 (0.003–0.2)	0.0135 (0.0075–0.04525)	0.37652	0.015 (0.012–0.079)	0.01 (0.007–0.031)	0.1928
>0.2	11/47 (23.4)	9/33 (27.27)	2/14 (14.29)	0.28702	1/5 (20)	1/9 (11.11)	0.6044
Cardiac troponin I, μg/L	0.24 (0.055–0.4775)	0.24 (0.1–0.4)	0.29 (0.02–0.9)	0.38233	0.045 (0.014–0.6925)	0.4 (0.25–5.25)	0.2669
>1.5	6/42 (14.29)	3/29 (10.34)	3/13 (23.08)	0.262	1/6 (16.67)	2/7 (28.57)	0.56294

*Data are median (IQR)or n/N (%). P-values were calculated by Fisher's exact test or Mann-Whitney U-test. CT, computerized tomography; Ag, antigen; IgM, immunoglobulin m; Ab, antibody; HBsAg, hepatitis B surface antigen; HCV-Ab, hepatitis C virus antibody; TP-Ab, treponema pallidum antibody; PH, pondus hydrogenii; PCO_2_, partial pressure of carbon dioxide; PO_2_, partial pressure of oxygen*.

In the first nucleic acid testing, 323 (65.25%) were confirmed positive for SARS-CoV-2. The leucocyte count in non-survivors (8.66 × 10^9^/L [IQR 7–12.335]) was significantly higher than that in mild and severe survivors. Lymphocytopenia is more common in the severe than in the mild (39.24 vs. 18.16%). 96.65% of patients experienced a decrease in eosinophil count. The level of D-dimer at admission was significantly higher in severe patients (0.8 mg/L [IQR 0.19–4.18] vs. 0.39 mg/L [IQR 0.16–0.98], *p* = 0.04076), and the non-survivors was significantly higher than the survivors (6.9 mg/L [IQR 1–32.47] vs. 0.46 mg/L [IQR 0.1625–1.63225, *p* = 0.00199). The alanine aminotransferase, lactate dehydrogenase and creatine kinase in the severe were significantly higher than those in the mild, and the non-survivors was more obviously, the difference was significant. The incidence of renal impairment was higher in the non-survivors. The incidence of arterial blood gas hypoxia and respiratory alkalosis on admission in the non-survivors was higher than that in the mild and the severe survivors. Three hundred and seventy-two people were tested for C-reactive protein (CRP) upon admission. Two hundred and eleven (56.72%) had CRP > 10 mg/L. The increase rate in the severe (85.51%) was significantly higher than that in the mild (50.17%). Two hundred and thirty-five patients were tested for procalcitonin (PCT) upon admission, and 100% patients in the non-survivors had elevated PCT. Patients in non-survivors had more laboratory abnormalities than those in mild and severe.

### Treatments During the Hospitalization

Two hundred and seventeen (41.49%) patients received respiratory support during hospitalization, of which 18 (4.2%) of mild patients received nasal catheter inhalation, as shown in Table 3. The respiratory support rate of the severe was higher than that of the mild, and the non-survivors all received mechanical ventilation treatment, of which six received non-invasive mechanical ventilation treatment and 11 received invasive mechanical ventilation treatment. Nine patients in the severe received ECMO treatment, and no one survived. Thirty-nine (52.7%) of the severe survivors were treated with CRRT, and only 5 (33.33%) of the non-survivors applied this technique. In terms of drug treatment, antiviral treatment was commonly used in each group. The severe had a higher proportion of antibiotics than the mild, and the non-survivors had a higher proportion of carbapenem and glycopeptide antibiotics than the survivors. One hundred and twelve (21.41%) received glucocorticoid therapy, and the non-survivors received a higher proportion of glucocorticoid therapy than the severe survivors (62.5 vs. 41.03%).

**Table 3:** Treatments during the hospitalization.

**Treatments**	**All patients (*N* = 523)**	**Disease severity**		**Clinical outcome in severe**	
		**Mild (*N* = 429)**	**Severe (*N* = 94)**	**Non-survivors (*N* = 16)**	**Survivors (*N* = 78)**
**Oxygen support**	217/523 (41.49)	152/429 (35.43)	65/94 (69.15)	15/16 (93.75)	50/78 (64.1)
Oxygen inhalation through nasal catheter	73/523 (13.96)	18/429 (4.2)	55/94 (58.51)	9/16 (56.25)	46/78 (58.97)
Usage time, days	10 (6–14.5)	10 (7–14)	7.5 (4–14.75)	3.5 (2–4.25)	11 (4–15.75)
High-flow oxygen	48/523 (9.18)	0/429 (0)	48/94 (51.06)	10/16 (62.5)	38/78 (48.72)
Usage time, days	6 (4–11)	NA (NA-NA)	6 (4–11)	3.5 (2–4.75)	8 (4.75–12.25)
Non-invasive mechanical ventilation	15/523 (2.87)	0/429 (0)	15/94 (15.96)	6/16 (37.5)	9/78 (11.54)
Usage time, days	6 (5–12)	NA (NA-NA)	6 (5–12)	4.5 (3.25–7.25)	7 (6–13)
Invasive mechanical ventilation	13/523 (2.49)	0/429 (0)	13/94 (13.83)	11/16 (68.75)	2/78 (2.56)
Usage time, days	2 (1.25–7.75)	NA (NA-NA)	2 (1.25–7.75)	2 (1–4)	13 (13–13)
ECMO	9/523 (1.72)	0/429 (0)	9/94 (9.57)	9/16 (56.25)	0/78 (0)
Usage time, days	7 (2–9)	NA (NA-NA)	7 (2–9)	7 (2–9)	NA (NA-NA)
**CRRT**	44/449 (9.8)	0/360 (0)	44/89 (49.44)	5/15 (33.33)	39/74 (52.7)
Adsorptive	12/44 (27.27)	0/0 (NA)	12/44 (27.27)	3/5 (60)	9/39 (23.08)
Usage time, times	3 (2–3)	NA (NA-NA)	3 (2–3)	2 (1.5–2.5)	3 (3–3)
Non-adsorptive	32/44 (72.73)	0/0 (NA)	32/44 (72.73)	2/5 (40)	30/39 (76.92)
Usage time, times	2 (2–2)	NA (NA-NA)	2 (2–2)	NA (NA-NA)	2 (2–2)
**Drug Treatment**
Antiviral treatment	497/523 (95.03)	408/429 (95.1)	89/94 (94.68)	14/16 (87.5)	75/78 (96.15)
Other	174/523 (33.27)	142/429 (33.1)	32/94 (34.04)	4/16 (25)	28/78 (35.9)
Immunotherapy	181/523 (34.61)	126/429 (29.37)	55/94 (58.51)	13/16 (81.25)	42/78 (53.85)
Methylprednisolone	103/523 (19.69)	61/429 (14.22)	42/94 (44.68)	10/16 (62.5)	32/78 (41.03)
Usage time, days	5 (3–6)	5 (3–6)	5 (3–6)	5 (3–6)	5 (3–6)
Prednisone	9/523 (1.72)	9/429 (2.1)	0/94 (0)	0/16 (0)	0/78 (0)
Usage time, days	6 (4–14)	6 (4–14)	NA (NA-NA)	NA (NA-NA)	NA (NA-NA)
Immunoglobulin	80/523 (15.3)	43/429 (10.02)	37/94 (39.36)	12/16 (75)	25/78 (32.05)
Usage time, days	4 (3–5)	4 (3–4.25)	4 (2–5)	2.5 (1.75–4.25)	4 (3–5)
Thymosin α	68/523 (13)	50/429 (11.66)	18/94 (19.15)	4/16 (25)	14/78 (17.95)
Usage time, days	8.5 (6–13.75)	8 (5–13)	11 (8–15)	9 (6.5–10)	11 (8.25–15.75)
Antibiotics	347/523 (66.35)	279/429 (65.03)	68/94 (72.34)	12/16 (75)	56/78 (71.79)
Quinolone	266/523 (50.86)	220/429 (51.28)	46/94 (48.94)	5/16 (31.25)	41/78 (52.56)
Penicillin	67/523 (12.81)	51/429 (11.89)	16/94 (17.02)	3/16 (18.75)	13/78 (16.67)
Cephems	99/523 (18.93)	72/429 (16.78)	27/94 (28.72)	6/16 (37.5)	21/78 (26.92)
Carbapenem	25/523 (4.78)	11/429 (2.56)	14/94 (14.89)	6/16 (37.5)	8/78 (10.26)
Glycopeptide	9/523 (1.72)	2/429 (0.47)	7/94 (7.45)	5/16 (31.25)	2/78 (2.56)
Tetracycline	11/523 (2.1)	3/429 (0.7)	8/94 (8.51)	2/16 (12.5)	6/78 (7.69)
Antifungai agents	26/523 (4.97)	6/429 (1.4)	20/94 (21.28)	10/16 (62.5)	10/78 (12.82)
Fluconazole	4/523 (0.76)	0/429 (0)	4/94 (4.26)	2/16 (12.5)	2/78 (2.56)
Voriconazole	11/523 (2.1)	1/429 (0.23)	10/94 (10.64)	6/16 (37.5)	4/78 (5.13)
Caspofungin	8/523 (1.53)	0/429 (0)	8/94 (8.51)	5/16 (31.25)	3/78 (3.85)
Anti-inflammatory treatment	198/523 (37.86)	148/429 (34.5)	50/94 (53.19)	11/16 (68.75)	39/78 (50)
Acetylcysteine Effervescent Tablets	38/523 (7.27)	27/429 (6.29)	11/94 (11.7)	1/16 (6.25)	10/78 (12.82)

*Data are median (IQR)or n/N (%). ECMO, extracorporeal membrane oxygenation; CRRT, continuous renal replacement therapy*.

### Death Prediction Model

**Figure 2:**
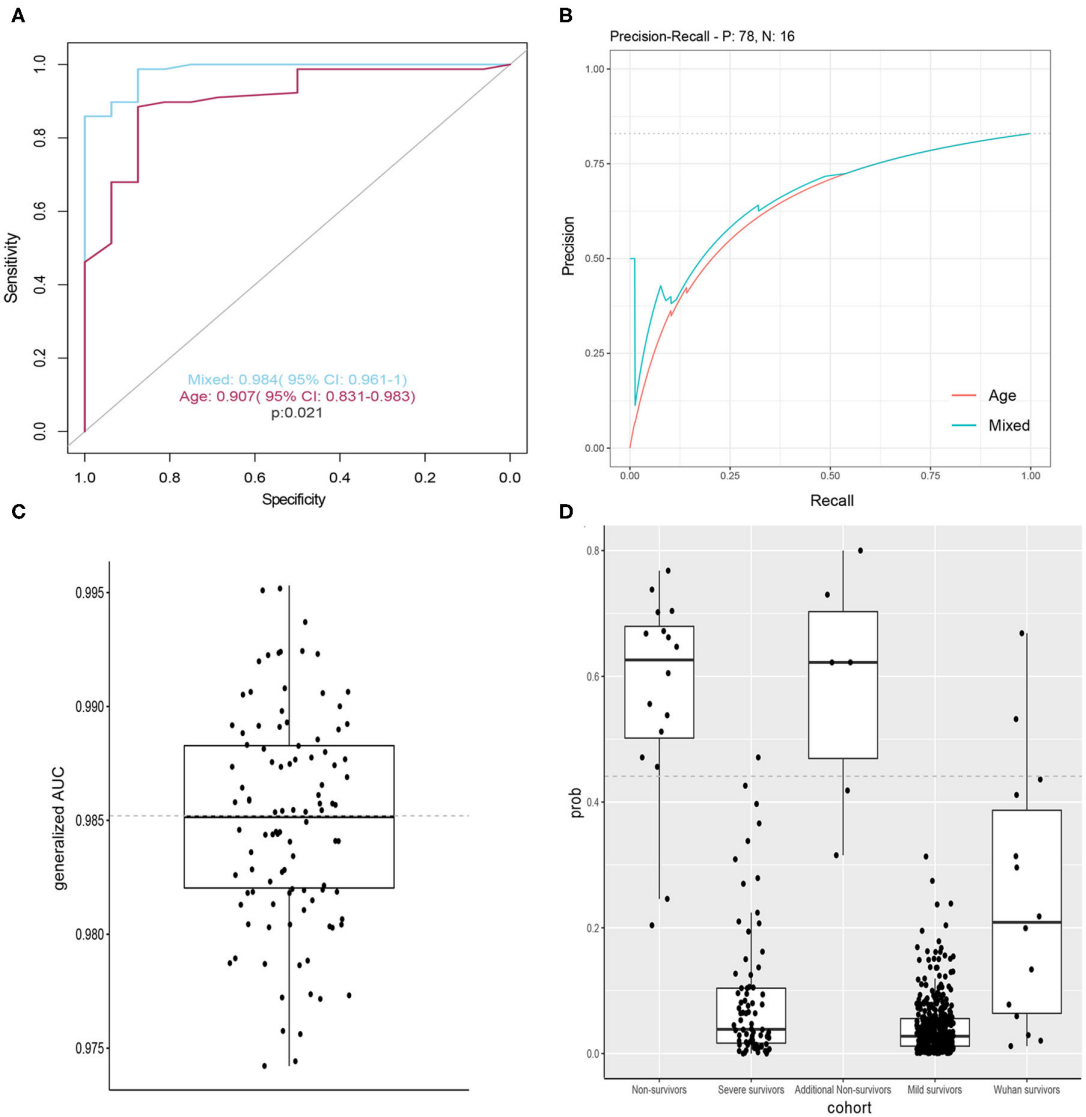
Models to predict death risk. **(A)** Performance of the classifiers using AUCs, significance determined by single sided AUC comparison by using bootstrap method with 10,000 permutations (boot. *n* = 10,000). **(B)** Precision-recall curves of models based on mixed features and age. **(C)** Distribution of generalized AUC by using bootstrap sampling (*n* = 100). **(D)** Boxplots showing distribution of death probabilities among different cohorts. horizon dashed line indicates selected threshold.

We constructed classification models to evaluate death risk for severe patients. Model performance was assessed by receiver operating characteristic (ROC) curve analysis using the area under the curve (AUC). In considering age is among leading risk factors for poor prognosis in several studies (3, 6, 7, 9–11), we firstly constructed models by using single age, which could achieve and AUC of 0.907 (95% CI 0.831–0.983) for death and alive severe COVID-19 patients. Mixed models constructed with combination of age, demographics, symptoms, and laboratory tests when firstly admitted to hospital had better performance (*p* = 0.021) and could achieved an AUC of 0.984 (95% CI 0.961–1) for death and alive severe COVID patients (Figures 2A,B). In considering fetal cases are with a small sample size, we randomly chose 40 samples from severe cases, then calculated the generalized AUC by using death probabilities and the median generalized AUC was 0.9852 (Figure 2C). Pulse oxygen, age, creatinine, creatine kinase, D-Dimer are the most important features (Table 4). We chose 0.441 as death prediction threshold (with 0.85 sensitivity and 0.987 specificity), then used six additional fatal cases (Henan), 429 mild cases and 14 cases (Wuhan) as independent validation cohort, and four in six death cases (0.67%) were assigned as death and majority of predicted death probabilities in the mild Henan cases and those Wuhan cases were below 0.441 (Figure 2D). Summary characteristics of six Henan additional fatal cases and 14 Wuhan cases and were outlined in Table 5.

**Table 4:** Importance of features in death risk prediction model.

**Feature**	**Mean decrease Gini**
Pulse oxygen saturation <90%	3.234
Age	3.025
Creatinine	1.907
Creatine Kinase	1.903
D-Dimer	1.787
Neutrophil percentage	1.292
Lactic dehydrogenase	1.274
Leucocyte count	1.207
Albumin	1.047
Time form illness onset to hospital admission	0.815
Glutamic-oxalacetic transaminase	0.737
Neutrophil count	0.731
Lymphocyte percentage	0.685
Respiratory rate >24 breaths per min	0.613
Prothrombin time	0.587
Blood urea nitrogen	0.562
Platelet	0.504
Direct bilirubin	0.486
C-reactive protein	0.405
Incubation period	0.388
Eosinophil percentage	0.264
Temperature	0.259
Chronic respiratory disease	0.207
Chronic obstructive pulmonary disease	0.202
Chest tightness	0.125
Diabetes	0.119
Coronary heart disease	0.116
Chest CT	0.095
Cardiovascular disease	0.072
Hypertension	0.064
Dyspnea	0.057
Cluster	0.050
Expectoration	0.031

*CT, computerized tomography*.

**Table 5:** Summary characteristics of six Henan additional fatal cases and 14 Wuhan cases.

**Feature**	**WH (n=14)**	**HN (N=6)**
Age	63.5 (45–75.5)	77 (65–78.5)
Time form illness onset to hospital admission	10 (7–14.75)	2.5 (1–7)
Breath	21.5 (20.25–22.75)	24.5 (23.25–25.75)
Cardiovascular disease	3/14 (21.43)	4/5 (80)
Diabetes	2/14 (14.29)	1/5 (20)
Coronary heart disease	2/14 (14.29)	1/5 (20)
Systolic pressure	128 (123.25–133)	157.5 (144.5–160)
Chest tightness	10/14 (71.43)	2/6 (33.33)
Pulse oxygen saturation	92.5 (90–94.75)	93.5 (89–95)
Chronic respiratory disease	1/14 (7.14)	2/5 (40)
Chronic obstructive pulmonary disease	1/14 (7.14)	2/5 (40)
Hypertension	5/14 (35.71)	4/5 (80)
Temperature	36.75 (36.5–36.88)	37 (36.58–37.43)
Sputum	2/14 (14.29)	4/6 (66.67)
Cluster	2/14 (14.29)	1/6 (16.67)
Dyspnea	7/14 (50.00)	1/6 (16.67)
Incubation period	0 (0–0)	8 (4–10.5)
White Blood Cell	5.95 (4.74–7.09)	8.04 (6.52–10.53)
Aspartate Aminotransferase	25 (23–30)	28.5 (18.5–50.5)
Lactic dehydrogenase	175.5 (153.5–235)	566.5 (294.25–876.25)
Blood urea nitrogen	6.13 (4.32–7.27)	10.46 (5.01–18.28)
Neutrophil percentage	69.4 (62.7–79.1)	92.35 (90.83–94.4)
Albumin	32.8 (30.2–37.6)	33.2 (29.38–35.75)
Creatinine	70 (60.2–103)	57 (42.36–67.25)
D-Dimer	0.71 (0.33–0.86)	40.31 (40.31–40.31)
Neutrophil count	4.33 (3.23–5.29)	7.33 (6.1–9.92)
Creatine Kinase	66.5 (50.25–115.25)	133.5 (66.25–197.75)
Platelet	235.5 (196.25–309)	110 (98–156.5)
Direct bilirubin	4.2 (3.1–6.1)	4.1 (3.1–13.5)
Lymphocyte percentage	18.6 (12.8–28)	5 (3.33–6.3)
Eosinophil percentage	0.6 (0–1)	0.1 (0.03–0.33)
C-reactive protein	23.35 (3.95–66.53)	55.25 (19.74–106.43)
Prothrombin time	11.5 (10.7–12.2)	14.2 (14.1–14.3)

*Data are median (IQR)or n/N (%)*.

## Discussion

Henan Province has a large population of 95.593 million people, bordering Hubei Province, China. As of April 1, 2020, there were 1,273 people confirmed COVID-19 in Henan, which was the second most in China outside Hubei Province. We collected data of 523 confirmed COVID-19 cases who had been discharged from 18 cities in Henan Province before February 20, 2020 and conducted statistical analysis. Our data showed that the main epidemiological characteristics of novel coronavirus pneumonia in Henan Province were import and cluster, which were similar to other provinces and cities outside Hubei in China. Among the 523 cases, there were 289 males (55.26%) and 234 females (44.74%). Other reports also showed a higher percentage of males (9, 12, 13), suggesting that males were more susceptible. Our study suggested that people of all ages were generally susceptible, with people aged 18–64 accounting for 87.96%, which was consistent with the Chinese CDC report (3). In our study, there were 16 fatal cases before February 20, 2020, and 87.5% of the deaths were ≥65 years old, with a median age of 71 years, while the median age for the mild and severe survivors was 42 and 50 years, respectively. The most common comorbidities in the non-survivors were hypertension (46.67%), coronary heart disease (33.33%), diabetes (33.33%), and COPD (33.33%). The average number of comorbidities in non-survivors was 1.94. Several studies about severe novel coronavirus pneumonia in China suggested that advanced age and comorbidities were high-risk factors for COVID-19 patients to develop into severe and death (10, 13, 14). In our study, advanced age was the biggest risk factor for death, which was consistent with that. A study from Italy involving 1,043 critically ill COVID-19 cases showed similar results, but male patients accounted for a higher proportion (82%) (9).

The median incubation period of the 523 cases in Henan Province was 5 days, and there was no significant difference between mild and severe. The median time from the onset of symptoms to hospitalization in the non-survivors was 8 days, and it was significantly longer than the severe survivors, suggesting that a delay in hospitalization might be one of the factors leading to death. Fever (88.74%), cough (62.3%), fatigue (39.58%), and expectoration (28.75%) were the most common symptoms. In spite of more symptoms, 60.87% of the severe and 80% of non-survivors had a temperature below 37.5°C at the time of admission. Zhong et al.'s study on 1,099 cases of COVID-19 also found that 52% of patients did not have fever when they became ill (12). The lack of fever symptoms made it difficult to identify COVID-19 patients and could also be one of the factors that caused a delay in visiting the doctor. Another study on refractory COVID-19 also found that the refractory pneumonia cases had a significantly lower fever incidence than the common pneumonia cases, suggesting that slow or poor response to SARS-CoV-2 was more likely to cause severe illness (15).

Compared with the mild and severe survivors, the non-survivors had higher leucocyte count, neutrophil percentage, D-dimer, LDH, BNP, and PCT levels, while the proportion of eosinophils, lymphocytes and albumin were lower, which was consistent with other studies. White blood cell count, neutrophil percentage and elevated PCT suggested that the non-survivors might be hospitalized with bacterial infection. Low albumin indicated that the patient was seriously depleted and the nutritional level was poor. D-dimer elevation had been confirmed in multiple studies as a high-risk factor for severe illness and death (10, 16, 17), which was consistent with our study. Chen et al.'s study found that in the non-survivors 56% had increased leucocyte count and 91% had lymphopenia, while in the severe survivors 4% had increased leucocyte count and 47% had lymphopenia (10). Zhang et al.'s study found that most COVID-19 cases combined with lymphopenia (75.4%) and eosinophilia (52.9%), and lymphopenia and eosinophilia were associated with disease severity (17). In our study, eosinophilia generally occurred in all cases, and there was no significant difference between the non-survivors and the severe survivors, but most of the eosinophils in the severe survivors returned to normal when discharged, while that of the non-survivors continued to decrease. Liu et al. also found that eosinophilia might be an indicator of disease improvement (18).

In the non-survivors, 100% of the patients had chest CT pneumonia area > 15% at admission, which was more severe in imaging than the mild and severe survivors. In terms of respiratory support, the rate of mechanical ventilation in the non-survivors was significantly higher than that in the mild and the severe survivors, which also suggested that the lung function of the non-survivors was more seriously impaired. In the non-survivors, the percentage of invasive mechanical ventilation was 68.75%, higher than other reports from Wuhan, China, but lower than those reported by the United States (71%) and Italy (88%), and Henan Province's mortality rate was also lower than that of the United States and Italy (9, 19). In addition to the aging factor, the fatal rate difference between Italy and Henan Province could be due to the fact that the number of COVID-19 cases in Henan province was relatively smaller and the medical resources were relatively more sufficient. Nine patients were applied with extracorporeal membrane oxygenation and technology (ECMO), but no one survived. Research showed application of ECMO could reduce mortality of patients with H1N1-related ARDS and MERS-related ARDS (20, 21), but there was no large-scale clinical report on the application of ECMO in the treatment of COVID-19, and its success rate is still unclear. In Yang et al.'s report, six patients applied ECMO with only one survived (13). Only a few cases were reported with successful ECMO treatment (22, 23). The recovery of lymphocyte count was the key factor for improvement of COVID-19. The application of ECMO destroyed lymphocytes and affected the function of lymphocytes. At the same time, it could cause IL-6 increase. This could be a reason for the low success rate of ECMO treatment. How to successfully apply ECMO in the treatment of COVID-19 still requires further research. 52.7% of patients in the critical severe survivors applied CRRT technology, while 33.33% patients in the non-survivors, suggesting that CRRT could help improve the prognosis of COVID-19. The application rate of glucocorticoids in the non-survivors was significantly higher than that in the mild and the severe survivors, which was consistent with other studies. Glucocorticoids had been widely used in SARS-CoV and MERS-CoV, but studies showed that the application of glucocorticoids prolonged the clearance time of virus and the probability of mental illness was significantly increased (24). Similarly, there was no evidence that glucocorticoids were beneficial to improve the prognosis of patients with COVID-19. Whether glucocorticoids can improve the prognosis of COVID-19 still requires long-term follow-up and further research.

In our study, some independent risk factors for death were found and we firstly developed a forest tree to accurately predict clinical outcomes of patients with COVID-19 based on combination of age, demographic features, symptoms and clinical tests at admission. Old age was the most important risk factor for poor prognosis of COVID-19 patients. The mixed model conducted by forest tree performed well in predicting survival and death, with AUC of 0.984 (95% confidence interval 0.961–1) for survival and death, which is helpful for further understanding and improve clinical strategies against COVID-19. We also found the predicted value was positively correlated with the severity of COVID-19. Of the 14 confirmed cases from Wuhan, seven were mild, seven were severe, 13 were cured and discharged, and one was referred to other hospital due to critical illness. In the death prediction model based on Wuhan data, those with a predicted value >0.3 were all critically ill, and the respiratory support treatment intensity was higher than the other 10 cases. The predictive value of the case transferred to other hospitals due to critical illness was 0.673, unfortunately we failed to follow up on the clinical outcome. The death prediction model we have established has also been validated in mild and six other fatal cases in Henan Province. The prediction of death for all mild survivors was below 0.3 and 4 in six death cases (66.67%) were assigned as death.

Mild patients have rare fetal cases thus we excluded mild cases in the death prediction models. Several studies have constructed models for early identification of cases at high risk of progression to severe COVID-19 (11) or improved prognosis (25). However, fatal cases were always rapid disease progression and died in hospitals in a short time, though we have plenty of medical support in Henan province. To the best of our knowledge, this is the first death prediction model for COVID-19 established by random forest. The model can accurately predict the prognosis of patients with COVID-19. Our study provided a new method for the evaluation of disease severity. Early identification of high-risk COVID-19 cases and early supportive therapy is critical to the prognosis.

There are some limitations of our study. Firstly, this is a retrospective study. There was incomplete documentation of the history, symptoms, or laboratory findings in some cases, even after trying to feedback and recollect. Secondly, as a retrospective and observational study, although this random forest model was validated in mild cases and additional fatal cases in Henan Province and 14 cases from Wuhan and showed good predictive effects, there were few validators outside Henan Province. Thirdly, imageology lacked objective judgment standards, and the investigators' judgment was subjective, which might lead to some bias.

## Data Availability Statement

The raw data supporting the conclusions of this article will be made available by the authors, without undue reservation. The datasets generated for this study can be found here: https://github.com/xiaoshubaba/COVID-Henan.

## Ethics Statement

The studies involving human participants were reviewed and approved by Ethics Committee from The First Affiliated Hospital of Zhengzhou University. Written informed consent for participation was not required for this study in accordance with the national legislation and the institutional requirements.

## Author Contributions

QZ, AX, and ZY made substantial contributions to conception, designed the study, had full access to all of the data in the study, take responsibility for the integrity of the data, and the accuracy of the data analysis. XM and AL drafted the manuscript, critically revised the manuscript for important intellectual content, and gave final approval for the version to be published. XM, AL, MJ, QS, and XA did the data analysis. XM, AL, MJ, QS, XA, YF, HLia, JC, HLi, JL, ZR, RS, GC, YZ, MC, LX, PJ, and YW collected the data and checked the data. All authors agree to be accountable for all aspects of the work in ensuring that questions related to the accuracy or integrity of any part of the work are appropriately investigated and resolved.

## Conflict of Interest

The authors declare that the research was conducted in the absence of any commercial or financial relationships that could be construed as a potential conflict of interest.
